# Varus alignment of the hip and knee 2 years after anterior cruciate ligament injury is associated with medial tibiofemoral osteoarthritis 3 years later

**DOI:** 10.1002/jeo2.70143

**Published:** 2025-01-03

**Authors:** Henrik Nilsson, Martin Englund, Richard Frobell, L. Stefan Lohmander, André Struglics, Per Swärd

**Affiliations:** ^1^ Department of Clinical Sciences Lund, Orthopaedics, Clinical and Molecular Osteoporosis Research Unit Faculty of Medicine Lund University Lund Sweden; ^2^ Department of Clinical Sciences Lund, Orthopaedics, Clinical Epidemiology Unit Faculty of Medicine Lund University Lund Sweden; ^3^ Department of Clinical Sciences Lund, Orthopaedics Faculty of Medicine Lund University Lund Sweden

**Keywords:** alignment, anterior cruciate ligament, hip knee ankle angle, neck‐shaft angle, osteoarthritis

## Abstract

**Purpose:**

To investigate if hip and knee alignment assessed 2 years after anterior cruciate ligament (ACL) injury is associated with compartment‐specific radiographic knee osteoarthritis (OA) 3 years later.

**Methods:**

An exploratory analysis was conducted in the knee ACL, nonsurgical versus surgical treatment (KANON) trial (ISRCTN84752559); 115 subjects with acute ACL injury were assessed at the 2‐year follow‐up; full‐limb images of the injured leg were acquired, and the neck‐shaft angle (NSA) and hip‐knee‐ankle angle (HKA) were measured. At the 5‐year follow‐up, weight‐bearing tibiofemoral and patellofemoral radiographs were obtained. Radiographs were graded according to the OA Research Society International Atlas and Radiographic OA was defined as approximating Kellgren & Lawrence grade 2 or worse. Analysis of covariance adjusting for sex, age, body mass index, randomization and partial meniscectomy recorded at the 2‐year follow‐up was performed.

**Results:**

In patients who had developed medial tibiofemoral OA at the 5‐year follow‐up, the NSA and the HKA at the 2‐year follow‐up were smaller (NSA, mean difference = −4.6° [95% confidence interval {CI} −7.9° to −1.1°]; HKA, mean difference = −2.3° [95% CI −4.2° to −0.4°]). No association was observed between the NSA or HKA at the 2‐year follow‐up and lateral tibiofemoral OA, nor patellofemoral OA at the 5‐year follow‐up.

**Conclusion:**

A smaller NSA and HKA angle of the ACL injured leg (i.e., more varus hip and varus knee alignment) 2 years after the injury was associated with medial tibiofemoral radiographic OA 3 years later.

**Level of Evidence:**

Level II exploratory post hoc analysis of an RCT.

**Abbreviations**
ACL: anterior cruciate ligament
BMI: body mass index
CI: confidence interval
GE: general electric
HKA: hip‐knee‐ankle angle
JSN: joint space narrowing
KANON: knee anterior cruciate ligament, nonsurgical versus surgical treatment
NSA: neck‐shaft angle
OA: osteoarthritis
OR: odds ratio
PF: patellofemoral
SD: standard deviation
SPSS: statistical package for the social sciences
TF: tibiofemoral

## INTRODUCTION

A major risk factor for knee osteoarthritis (OA) development and progression is when the load on the joint surfaces exceeds the mechanical properties of the cartilage [11, 15]. A high joint load can be caused by a high body mass index (BMI) [32], redistribution of the joint loading after a meniscal tear and/or meniscectomy [9] or as a result of altered loading patterns in the case of an anterior cruciate ligament (ACL) injury [7]. As such, ACL injury is a well‐known risk factor for both tibiofemoral (TF) and patellofemoral (PF) OA in younger individuals, that is, posttraumatic OA [21, 22]. The risk for posttraumatic OA increases when the meniscus is injured in addition to the ACL, and especially after meniscectomy [7].

In studies of nontraumatic knee OA, anatomical variations in the hip and in knee alignment have been reported to be associated with compartment‐specific OA [28]. The mechanical alignment of the knee, measured as the hip‐knee‐ankle angle (HKA), affects load transmission in the TF joint [10] and alters patella kinematics and contact forces [6]. There is ample evidence that varus and valgus malalignment of the knee is associated with knee OA progression of the medial and lateral TF compartment, respectively [28]. Varus malalignment has furthermore been associated with the development of medial TF OA [5, 29], whereas no such association was reported for valgus alignment and lateral TF OA development. No association has been found between knee alignment, changes in knee cartilage volume and TF OA development [17, 34]. It has been suggested that differences in hip and pelvic geometry can explain sex‐related differences in OA, with an increased incidence of lateral TF OA compared to medial TF OA in females compared to males [4]. Convincing evidence that posttraumatic OA development after ACL injury is associated with mechanical alignment of the injured leg has not been documented [30], and the relationship between hip anatomy and knee OA development after ACL injury is unknown.

**Figure 1:**
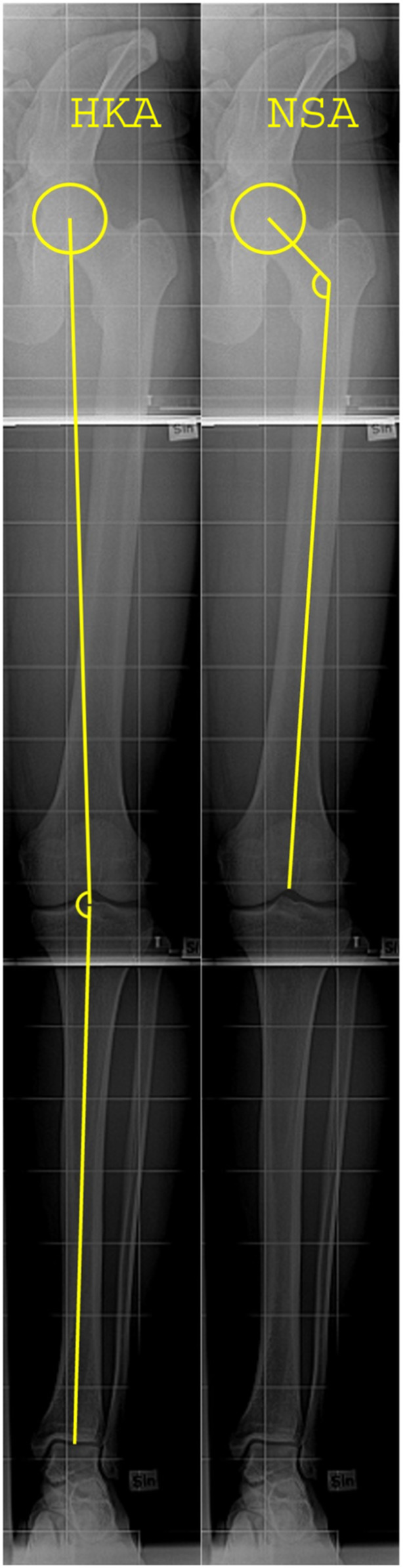
A schematic illustration of the HKA and the NSA measurements. Full‐limb radiographs were obtained of the injured leg with the radiographed leg in weight‐bearing and in slight knee flexion. The HKA was assessed by drawing a line from the centre of the femoral head to the centre of the tibial spines. An additional line was drawn from the centre of the trochlea of talus to the centre of the tibial spines. The HKA angle was defined as the medial angle at the intersection of the two lines. The NSA was defined as the angle between the femoral neck axis and the femoral long axis. The femoral neck axis was defined as the line between the centre of the femoral head and the femoral neck centre. The two radiographs are from one of the patients in the knee anterior cruciate ligament, nonsurgical versus surgical treatment study. HKA, hip‐knee‐ankle angle; NSA, neck‐shaft angle.

The aim of the present study was to investigate if hip and knee alignment assessed 2 years after ACL injury was associated with compartment‐specific OA of the injured knee 3 years later (Figure 1). We hypothesized that a higher neck‐shaft angle (NSA) and valgus alignment of the ACL injured knee would be associated with the development of lateral TF OA, whereas a smaller NSA and varus alignment would be associated with medial TF OA. In a secondary analysis, we aimed to assess the association between alignment at the 2‐year follow‐up and partial meniscectomy performed up to the 2‐year follow‐up.

## METHODS

Active young adults (18–35 years, 26% women) with an ACL injury within four weeks before examination were recruited for the knee ACL, nonsurgical versus surgical treatment (KANON) trial (ISRCTN84752559). Exclusion criteria were previous ACL injury to the knee, total collateral ligament ruptures, extensive meniscal fixation, professional athletes with a Tegner score of 10 and inactive people with a Tegner score <5. All patients were recruited from Skåne, the southern region of Sweden. A total of 121 patients were included and 62 were randomized to undergo rehabilitation with early ACL reconstruction and 59 to undergo rehabilitation and optional delayed ACL reconstruction [13]. Of the 59 to undergo rehabilitation and optional delayed ACL reconstruction a total of 23 underwent ACL reconstruction, on average 347 ± 124 days from baseline [13].

At the 2‐year follow‐up, full‐limb radiographs were obtained of the index leg of all the participants, with the radiographed leg in weight‐bearing and in slight knee flexion. The radiographs were obtained using a General Electric Prestige II (General Electric Medical Systems). The HKA and the NSA were measured using the RadiAnt digital imaging and communications in medicine Viewer (version 1.9.16.7446, Medixant) with an accuracy of 0.1° [3]. The HKA was assessed by drawing a line from the centre of the femoral head to the mid‐distance of the tibial spines. An additional line was drawn from the centre of the trochlea of talus to the centre of the tibial spines. The HKA angle was defined as the medial angle at the intersection of the two lines (Figure 1) [16, 19]. The NSA was defined as the angle between the femoral neck axis and the femoral long axis, with the femoral neck axis defined as the line between the centre of the femoral head and the femoral neck centre. The femoral long axis was assessed by drawing a line through two points; one in the centre of the femoral condyles and the other in the middle of the femoral shaft, just below the lesser trochanter (Figure 1) [3].

At the 5‐year follow‐up, radiographs of the injured knee were performed. Axial view of the PF joint was acquired as previously described, according to Ahlbäck [2, 14]. TF radiographs were acquired using a modified version of the Lyon‐Schuss projection, where the subject stands with equal weight bearing on each leg [8, 14]. Radiographs were graded according to the OA research society international atlas and radiographic OA was defined based on any of the following three criteria in either of the two TF compartments or in the PF compartment that was present: (a) joint space narrowing (JSN) grade ≥2; (b) the sum of the osteophyte compartment grades ≥2 or (c) JSN grade of 1 together with a grade 1 osteophyte within the same compartment. These criteria approximate radiographic knee OA grade ≥2 according to the Kellgren and Lawrence scale [9, 20].

At the 5‐year follow‐up, 116 of the 121 individuals included in the KANON trial underwent full‐limb radiography of the injured leg. Because of bad film quality, no measurements were made on one of the radiographs, leaving measurements of 115 ACL‐injured legs. The 115 patients with full‐limb radiographs of the injured leg were included in the present analysis [14]. To test intrarater variability of HKA and NSA assessments, 20 radiographs were re‐measured (1 month between measurements) and the intraclass correlation was 0.98 (95% confidence interval [CI] 0.97–0.99) for the HKA and 0.95 (95% CI 0.86–0.98) for the NSA.

All 115 subjects completed the knee injury and OA outcome score for patient‐assessed outcomes at the 5‐year follow‐up to assess how the patient perceived pain, other symptoms, function in sports and recreation and knee‐related quality of life, during the previous week [27].

### Statistics

Statistical package for the social sciences (SPSS) statistical software (version 22, SPSS Inc.) was used for all analyses. Continuous variables were reported as mean (SD) and differences between groups were tested with the Student's *t* test. All continuous variables were normally distributed. Differences between groups in dichotomous variables were tested with the *χ* ^2^ test. Analysis of covariance was used to test those variables which were significantly associated with OA in the crude analysis. Adjustments were made for sex, age, BMI, randomization, partial medial meniscectomy and partial lateral meniscectomy of the injured knee. In a secondary analysis, the knees were categorized as varus (HKA‐angle ≤ 178°), neutral (HKA‐angle 179–181°) and valgus (HKA‐angle ≥ 182°) and logistic regression was used to test how varus and valgus alignment in relation to neutral alignment 2 years after ACL injury related to the prevalence of OA 5 years after ACL injury.

## RESULTS

A total of 115 patients were included in the study (Table 1). The patients had a mean BMI of 24.1 and a mean age of 26.2 years (26% were female); 20% of the patients underwent partial medial meniscectomy, 30% underwent partial lateral meniscectomy and 17% underwent meniscal fixation before the 2‐year follow‐up. Neither the HKA nor the NSA was statistically associated with randomization to undergo rehabilitation with early ACL reconstruction versus undergoes rehabilitation and optional delayed ACL reconstruction (Supporting Information S1: Table S1).

**Table 1:** Patient characteristics at baseline.

All patients, *n*	115
Women, *n* (%)	30 (26)
Age, mean years (SD)	26.2 (5.0)
Body mass index, mean kg/m^2^ (SD)	24.1 (2.9)
Randomized to ACL reconstruction, *n* (%)	59 (51)
Partial medial meniscectomy, *n* (%)	23 (20)
Partial lateral meniscectomy, *n* (%)	34 (30)
Meniscal fixation, *n* (%)	20 (17)

Abbreviations: ACL, anterior cruciate ligament; SD, standard deviation.

### Association between NSA and HKA angle 2 years after ACL injury with OA 5 years after ACL injury

In patients who had developed medial TF OA at the 5‐year follow‐up after ACL injury, the HKA angle of the injured leg at the 2‐year follow‐up was 176.4° compared to 178.7° in patients who had not developed medial TF OA; mean difference, −2.3° (95% CI −4.2° to −0.4°) (Table 2). In patients who had developed medial TF OA, the NSA of the injured leg at the 2‐year follow‐up was 124.7° compared to 129.3° in patients who had not developed medial TF OA; the mean difference was −4.6° (95% CI −7.9° to −1.1°). In the adjusted analysis model, the HKA and NSA angles remained statistically significantly different between patients who had developed medial TF OA and those that had not developed medial TF OA (Table 2). No statistically significant association was observed between the NSA or HKA at the 2‐year follow‐up and lateral TF OA, nor PF OA, at the 5‐year follow‐up (Table 2).

**Table 2:** Compartmental radiographic OA at 5 years after anterior cruciate ligament injury in relationship to NSA and HKA at 2 years after injury.

	OA at the medial tibiofemoral compartment		*p* Values	Adjusted *p* Values
	Yes, *n* = 9	No, *n* = 106		
HKA	176.4° (2.9°)	178.7° (2.8°)	0.02	0.04
NSA	124.7° (2.8°)	129.3° (5.0°)	<0.01	0.02

	OA at the lateral tibiofemoral compartment		*p* Values	Adjusted *p* Values
	Yes, *n* = 7	No, *n* = 108		
HKA	178.2° (2.9°)	178.5° (2.9°)	0.80	0.63
NSA	128.6° (6.7°)	129.0° (4.9°)	0.86	0.76

	OA at the patellofemoral compartment		*p* Values	Adjusted *p* Values
	Yes, *n* = 22	No, *n* = 93		
HKA	178.2° (3.5°)	178.5° (2.7°)	0.63	0.93
NSA	128.1° (3.6°)	129.1° (5.3°)	0.27	0.73

*Note*: HKA and NSA degrees are presented as mean (SD). Differences between groups were tested with the Student's *t* test and using analysis of covariance adjusting for sex, body mass index, randomization and partial medial and lateral meniscectomy.

Abbreviations: HKA, hip‐knee‐ankle angle; NSA, neck‐shaft angle; OA, osteoarthritis; SD, standard deviation.

#### The association between alignment and meniscectomies at the 2‐year follow‐up

Of the 115 patients included in the present study, 23 patients (20%) underwent a partial meniscectomy of the medial meniscus, and 34 patients (30%) underwent a partial meniscectomy of the lateral meniscus in the ACL injured knee between baseline and the 2‐year follow‐up. The HKA was not statistically different (*p* = 0.27) between subjects that had undergone a medial partial meniscectomy (mean, 177.2°; standard deviation [SD], 3.1°) and subjects that did not have a medial partial meniscectomy (mean, 177.9°; SD, 2.9°). The HKA was not statistically different (*p* = 0.23) between subjects that had undergone a lateral partial meniscectomy (mean, 177.3°; SD, 2.6°) and subjects that did not have a lateral partial meniscectomy (mean, 178.0°; SD.3.1°). Also, the NSA was not statistically different (*p* = 0.43) between subjects that had undergone a medial partial meniscectomy (mean, 128.7°, SD.4.7°) and subjects that did not have a medial partial meniscectomy (Mean 129.6°; SD, 5.2°). The NSA was not statistically different (*p* = 0.51) between subjects that had undergone a lateral partial meniscectomy (mean, 129.0°; SD, 6.0°) and subjects that did not have a lateral partial meniscectomy (mean, 129.7°; SD, 4.8°).

#### Varus, neutral and valgus limb alignment and the association with knee OA

In a secondary analysis, using HKA measurements from the 2‐year follow‐up, patients were characterized into varus, neutral or valgus knee alignment.

Of 64 patients with varus knees, 15 had TF OA (nine medial and six lateral) and 12 had PF OA of the injured knee at the 5‐year follow‐up; none of the 30 patients with neutral alignment had OA in the TF joint and 7 had PF OA; one of the 21 patients with valgus knees had lateral TF OA of the injured knee and three had PF OA (Table 3). Compared to neutral alignment, there was no statistically significant association between varus or valgus alignment at the 2‐year follow‐up and TF or PF OA at the 5‐year follow‐up (Table 3).

**Table 3:** Relation between knee alignment of the injured leg 2 years after ACL injury and compartment‐specific OA 3 years later.

	Alignment			Varus vs. neutral		Valgus vs. neutral	
	Varus (<179°) *n* = 64	Neutral (179°–81°) *n* = 30	Valgus (>181°) *n* = 21	OR (95% CI)	*p* Value	OR (95% CI)	*p* Value
Medial TF OA	9 (14%)	0 (0%)	0 (0%)	4.9 (0.6–40.6)	0.14	No data	No data
Lateral TF OA	6 (9%)	0 (0%)	1 (5%)	3.1 (0.4–27.0)	0.30	1.5 (0.1–25.4)	0.78
PF OA	12 (19%)	7 (23%)	3 (14%)	0.7 (0.3–2.2)	0.60	0.5 (0.1–2.4)	0.43

Abbreviations: ACL, anterior cruciate ligament; CI, confidence interval; OA, osteoarthritis OR: odds ratio; PF, patellofemoral; TF, tibiofemoral.

### The association between alignment and symptoms at 5 years

Neither NSA nor HKA of the ACL‐injured knee measured at 2 years was statistically significantly associated with KOOS outcomes at the 5‐year follow‐up (Supporting Information S1: Tables S2 and S3).

## DISCUSSION

In subjects, who have had an acute ACL rupture 2 years earlier, we found an association between a smaller NSA and HKA (more varus) of the ACL injured leg and development of radiographic medial TF OA 3 years later. We found no statistically significant association between alignment and the risk of lateral TF OA or PF OA.

Injury to the ACL is associated with an increased risk of posttraumatic OA [14, 21]. Posttraumatic OA after ACL injury is multifactorial. Factors such as meniscal injury, damage to the TF cartilage and older age at the time of surgery have been reported to associate with developing posttraumatic OA [21, 22]. Regarding ACL reconstruction and posttraumatic OA, randomized controlled trials have suggested no essential differences in radiographic OA between those with or without surgical reconstruction [23, 26].

The hip geometry affects the hip abductor lever arm and moment, where a short lever arm (coxa valga) is associated with a reduced abductor moment, less hip abductor strength, increased dynamic knee valgus and increased lateral TF loads during gait. Contrarily, a long lever arm (coxa vara) will increase the adduction moment at the knee and medial TF loads during gait [1, 16, 19]. In line with their hypotheses, Boissonneault et al. reported that knees with lateral OA had a larger NSA, whereas knees with medial OA had smaller NSA. The NSA influences the abductor angle and the abductor lever arm, thereby also affecting the force generated by abductors and adductors at the hip and loads at the knee during gait [4]. A large NSA and thus a shorter abductor lever arm of the hip leads to a decreased mechanical advantage of the abductor muscles [4]. This can lead to the fact that hip adduction is promoted and the dynamic knee alignment changes to valgus, decreasing the knee adduction moment, thereby increasing lateral TF loads and promoting lateral OA of the knee. Also, it was suggested that a shorter lever arm leads to an imbalance in muscular activity during gait. To compensate for the anatomical prerequisites, the abductor muscles and tensor fascia lata passing the knee laterally during loading increase their activity trying to compensate for the shorter lever arm, thus increasing the force applied on the lateral compartment [33]. With a smaller NSA and thus a longer lever arm, it was speculated that hip abduction is promoted, and the dynamic knee alignment changes to varus, increasing the knee adduction moment, thereby increasing medial TF loads and promoting medial OA of the knee [33]. In the literature, strengthening of the hip abductors is shown to decrease pain and improve functional scores in patients with medial TF OA patients [31].

Varus alignment of the knee has been proposed as a risk factor for posttraumatic OA after ACL injury [30]. Compared to neutral alignment of the knee, varus and valgus alignment shifts the load distribution of the joint where increasing varus alignment leads to increasing loads of the medial compartment and increasing valgus alignment leads to increasing loads of the lateral TF compartment [4]. This, in combination with altered loading of TF and PF cartilage caused by the meniscal damage/meniscectomies and decreased stability in anterior‐posterior and internal‐external rotation can cause increased loads on surfaces previously not exposed to these forces, thereby increasing the risk of OA development [7, 10]. Regarding nontraumatic OA, both knee alignment and hip geometry have been reported to affect the risk of OA development and progression, by affecting the compartment‐specific loads in the TF and PF joints [28].

In the present study, we found that the HKA angle was associated with the development of radiographic TF OA of the medial compartment, but not with TF OA of the lateral compartment. These findings are somewhat different from what has been observed in nontraumatic OA, where valgus alignment is a risk factor for lateral TF OA development [5, 29]. When categorizing patients as varus, neutral and valgus aligned, we found that all patients with medial TF OA at the 5‐year follow‐up had varus alignment at the 2‐year follow‐up. Interestingly, none of the patients of the present study with neutral alignment had any radiographic signs of TF OA. Nevertheless, there was no statistically significant difference between the groups regarding the risk of compartment‐specific OA (varus vs. neutral and valgus vs. neutral). The low number of patients who had developed medial‐ and lateral TF OA at the 5‐year follow‐up (*n* = 9 for medial and *n* = 6 for lateral) indicate that those results may be attributable to a type II error, suggesting that larger‐sized future studies on this topic are warranted. Due to the exploratory nature of the present study, no prestudy power calculations were performed.

To sum up, preexisting varus alignment of the hip and knee resulting in higher medial TF loads could alter ACL injury (regardless of reconstruction), associated instability and altered cartilage loading lead to a rapid development of medial TF OA.

PF loads may also be affected by knee alignment and hip geometry [25]. However, in the present study, we found no such associations. Previous studies have suggested that subjects with a varus knee alignment are twice as likely to develop medial PF OA [6].

One limitation to our study is the low prevalence of compartment‐specific TF OA and PF OA, suggesting a high risk of statistical type II errors. Also, investigating the HKA angle of the ACL‐injured knee, measured at the two‐year follow‐up is another limitation, since several factors associated with the ACL injury could alter the HKA angle of the injured leg in varus or valgus direction [18]. Factors such as meniscal pathology, bone attrition, osteophytes and ligament damage may in addition to cartilage loss contribute to the knee alignment [18]. Hence, early OA changes which may have developed already 2 years after the ACL injury could affect knee alignment, thereby biasing our findings. As regards the NSA, it decreases from around 160° at birth to an angle of around 130° ahead of skeletal maturity [12]. The radiographic assessment of the NSA can be influenced by the rotation of the hip. Without correction for the rotation of the hip, there is a 1° margin of error in the NSA [3]. The NSA and HKA were measured on full‐limb radiographs using a standardized protocol, hence limiting the risk of major discrepancies in hip rotation between patients. To certify that the radiograph was acquired with the knee in a straight frontal view, fluoroscopic guidance was used in the lateral view to ascertain that the posterior aspect of the femoral condyles was aligned. Previous investigations have shown that, with regard to the femoral transepicondylar and anterior‐posterior axes, the posterior femoral condylar tangent is internally rotated 3‐6° [24]. Hence, the full‐limb radiograph of the hip, in a view perpendicular to the posterior femoral tangent, will be in approximately 3–6° of hip external rotation. Because of the low number of subjects with OA, it was not possible to separately investigate men and women.

The strengths of our study include the use of standardized full‐limb radiographs to measure the HKA and NSA, the prospective study design and the low loss to follow‐up.

## CONCLUSION

We found that a smaller NSA and HKA of the ACL injured leg 2 years after injury was associated with the development of radiographic medial TF OA 3 years later. These findings indicate that a small NSA and HKA by leading to increased medial TF loads may increase the risk of medial TF OA development after ACL injury, regardless of ACL reconstruction or not.

## AUTHOR CONTRIBUTIONS

*Literature review, statistical analysis and manuscript writing/editing*: Henrik Nilsson. *Literature review, interpretation of data and manuscript writing/editing*: Martin Englund. *Data collection, interpretation of data and manuscript writing/editing*: Richard Frobell. *Data collection, literature review and manuscript writing/editing*: L. Stefan Lohmander. *Interpretation of data and manuscript writing/editing*: André Struglics. *Study design, literature review, statistical analysis and manuscript writing/editing*: Per Swärd. The final manuscript was read and approved by all authors.

## CONFLICT OF INTEREST STATEMENT

The authors declare no conflicts of interest.

## ETHICS STATEMENT

Our study is a post hoc analysis of an RCT, the KANON trial (ISRCTN84752559). The Lund University ethics committee approved the study, all participants signed informed consent.

## Supporting information

Supporting information.

## Data Availability

Patient data can only be made available in accordance with GDPR and ethics permission requirements. Further inquiries can be made to the corresponding author.
