# Supplementary material for: Varus alignment of the hip and knee 2 years after anterior cruciate ligament injury is associated with medial tibiofemoral osteoarthritis 3 years later
Source: J Exp Orthop. 2025 Jan 3;12(1):e70143. doi: 10.1002/jeo2.70143 (PMC11696252; doi:10.1002/jeo2.70143)
Supplement: Supplementary file 1 — Supporting information. [file JEO2-12-e70143-s001.docx]

**Supplementary data**

**Varus alignment of the hip and knee two years after anterior cruciate ligament injury is associated with medial tibiofemoral osteoarthritis three years later**

**Supplementary contents**

Table S1, page – 2

Table S2, page – 3

Table S3, page – 4

**Table S1.** Difference in NSA and HKA between patients randomized to early ACL reconstruction and optional delayed treatment. Differences between groups were tested with the Student’s t-test. No differences between the two groups were found.

|  | **Randomized to early ACL reconstruction** | | |  |
| --- | --- | --- | --- | --- |
|  | **Yes (n=59)** | **No (n=56)** |  | |
|  | Mean (SD) | Mean (SD) | P values | |
| HKA | 178.4° (2.8°) | 178.5° (3.0°) | 0.81 | |
| NSA | 128.6° (4.9°) | 129.3° (5.2°) | 0.43 | |

NSA, neck-shaft angle; HKA, hip knee ankle angle; ACL, anterior cruciate ligament; SD, standard deviation.

**Table S2.** Association between HKA or NSA versus KOOS variables. The correlation was calculated with Spearman’s Rho. There were no correlations between HKA, NSA or any of the KOOS variables.

| **HKA** |  |  |  |
| --- | --- | --- | --- |
|  |  | r_S_ | P values |
|  | Symptoms | -0.001 | 0.991 |
|  | Pain | 0.010 | 0.920 |
|  | ADL | -0.056 | 0.557 |
|  | Sports | -0.079 | 0.402 |
|  | QoL | 0.001 | 0.995 |
|  | KOOS4 | -0.031 | 0.743 |
| **NSA** |  |  |  |
|  |  | r_S_ | P values |
|  | Symptoms | 0.114 | 0.227 |
|  | Pain | 0.130 | 0.166 |
|  | ADL | 0.147 | 0.118 |
|  | Sports | 0.076 | 0.423 |
|  | QoL | -0.015 | 0.875 |
|  | KOOS4 | 0.057 | 0.547 |

ADL, activities of daily living; HKA, hip-knee-ankle angle; KOOS, knee osteoarthritis outcome score; NSA, neck-shaft angle; QoL, quality of life; Sports, sports and recreation.

**Table S3.** Patient reported outcomes at the 5-year follow-up versus hip alignment 2 years after ACL injury. Before analysis, knee alignment was characterized into varus, neutral and valgus based on the HKA angle. A HKA <179° was defined as varus, a HKA 179°-181° was defined as neutral and a HKA >181° as valgus. Group differences were tested using the Student’s T-test. Patients with neutral alignment had higher KOOS scores in all KOOS variables (compared to patients with varus and valgus), however none were statistically significantly parted.

|  | **Alignment** | | | **Mean between group differences** | | | |
| --- | --- | --- | --- | --- | --- | --- | --- |
|  | **Varus**  **(****<179^o^)**  **n=64** | **Neutral**  **(179^o^ -181^o^)**  **n=30** | **Valgus (>181^o^)**  **n=21** | **Varus vs Neutral** | **P value** | **Valgus vs Neutral** | **P value** |
| **Patient reported outcomes at 5 years** | | | | | | | |
| Mean (95% CI) KOOS | | | | | | | |
| KOOS4 | 79 (75-84) | 84 (79-89) | 77 (69-85) | -5 (-12 to 2) | 0.19 | -6 (-15 to 3) | 0.17 |
| Pain | 90 (87-93) | 93 (89-97) | 90 (84-95) | -3 (-9 to 2) | 0.21 | -3 (-10 to 3) | 0.35 |
| Symptoms | 84 (80-88) | 84 (78-91) | 84 (77-90) | -1 (-8 to 7) | 0.88 | -1 (-11 to 9) | 0.84 |
| ADL | 95 (93-98) | 97 (95-99) | 95 (91-99) | -2 (-5 to 2) | 0.40 | -2 (-7 to 2) | 0.29 |
| Sports | 76 (70-82) | 83 (78-88) | 70 (57-82) | -7 (-15 to 1) | 0.10 | -10 (-25 to 4) | 0.14 |
| QOL | 67 (62-76) | 76 (69-82) | 64 (54-75) | -8 (-17 to 1) | 0.07 | -10 (-22 to 1) | 0.08 |

HKA, hip-knee-ankle angle; NSA, neck-shaft angle; KOOS, knee osteoarthritis outcome score; QoL, quality of life; CI, confidence interval.
